# Precise in-frame integration of exogenous DNA mediated by CRISPR/Cas9 system in zebrafish

**DOI:** 10.1038/srep08841

**Published:** 2015-03-05

**Authors:** Yu Hisano, Tetsushi Sakuma, Shota Nakade, Rie Ohga, Satoshi Ota, Hitoshi Okamoto, Takashi Yamamoto, Atsuo Kawahara

**Affiliations:** 1 Laboratory for Developmental Gene Regulation, Brain Science Institute, RIKEN, 2-1 Hirosawa, Wako, Saitama, 351-0198, Japan; 2 Molecular Genetics Laboratory, Department of Mathematical and Life Sciences, Graduate School of Science, Hiroshima University, 1-3-1 Kagamiyama, Higashi-Hiroshima, Hiroshima, 739-8526, Japan; 3 Laboratory for Developmental Biology, Center for Medical Education and Sciences, Graduate School of Medical Science, University of Yamanashi, 1110 Shimokato, Chuo, Yamanashi, 409-3898, Japan

## Abstract

The CRISPR/Cas9 system provides a powerful tool for genome editing in various model organisms, including zebrafish. The establishment of targeted gene-disrupted zebrafish (knockouts) is readily achieved by CRISPR/Cas9-mediated genome modification. Recently, exogenous DNA integration into the zebrafish genome via homology-independent DNA repair was reported, but this integration contained various mutations at the junctions of genomic and integrated DNA. Thus, precise genome modification into targeted genomic loci remains to be achieved. Here, we describe efficient, precise CRISPR/Cas9-mediated integration using a donor vector harbouring short homologous sequences (10–40 bp) flanking the genomic target locus. We succeeded in integrating with high efficiency an exogenous *mCherry* or *eGFP* gene into targeted genes (*tyrosinase* and *krtt1c19e*) in frame. We found the precise in-frame integration of exogenous DNA without backbone vector sequences when Cas9 cleavage sites were introduced at both sides of the left homology arm, the eGFP sequence and the right homology arm. Furthermore, we confirmed that this precise genome modification was heritable. This simple method enables precise targeted gene knock-in in zebrafish.

Artificial site-specific nucleases such as zinc-finger nucleases (ZFNs), transcription activator-like nucleases (TALENs) and RNA-guided nucleases (RGNs) have become fundamental tools for genome editing in various model organisms, including zebrafish^1^,^2^,^3^,^4^,^5^. Both ZFNs and TALENs are chimeric proteins fusing the DNA-recognising domain and the *Fok*I nuclease catalytic domain, and they function as dimers. Their activity leads to the production of DNA double-strand breaks (DSBs) at a targeted genomic locus. RGNs based on the clustered regularly interspaced short palindromic repeats (CRISPR)/CRISPR-associated (Cas) 9 consist of two components: guide RNA (gRNA) and Cas9 nuclease^6^,^7^,^8^. These nucleases induce DSBs in a targeted genomic locus that can be repaired via genome maintenance mechanisms such as non-homologous end joining (NHEJ), microhomology-mediated end joining (MMEJ) and homologous recombination (HR)^9^,^10^,^11^,^12^. The NHEJ repair system directly connects the broken ends with insertion and/or deletion (indel) mutations at high frequency, leading to frameshift-mediated gene disruption. Once artificial site-specific nucleases having substantial activity have been designed, targeted gene-knockout zebrafish can be established relatively easily^13^,^14^.

Precise integration of an exogenous DNA sequence into a targeted genomic locus has been highly desired. Using single-stranded oligodeoxynucleotides (ssODNs) with short homology arms, several groups have achieved precise genome modifications with high frequency^2^,^15^,^16^,^17^,^18^. In this method, however, the length of the integrated sequence is limited to short fragments such as restriction enzyme recognition sites and *loxP*. A double-strand DNA donor harbouring long homology arms can induce highly precise modifications that are able to integrate much longer exogenous DNA, although donor construction is time-consuming^19^,^20^.

Recently, a novel method capable of integrating long exogenous DNA fragments into the genome at high frequency was reported^21^,^22^,^23^. In this method, using a donor vector containing recognition sequences of artificial site-specific nucleases, a targeted genomic locus and a donor vector are simultaneously cleaved and connected to each other, presumably through NHEJ, resulting in the integration of the entire donor vector into the targeted genomic locus. Because of the NHEJ repair system, integration directions become random and various types of indel mutations are introduced at the junction, making it difficult to construct a chimeric protein fusing endogenous and exogenous genes by in-frame connection. Here, we report the efficient and precise integration of exogenous DNA into the zebrafish genome using a donor vector harbouring gRNA target sequences and short homologous sequences flanking the genomic target locus.

## Results

### Introduction of short homology arms into the donor vector leads to the precise integration of exogenous DNA into the zebrafish genome

**Figure 1:**
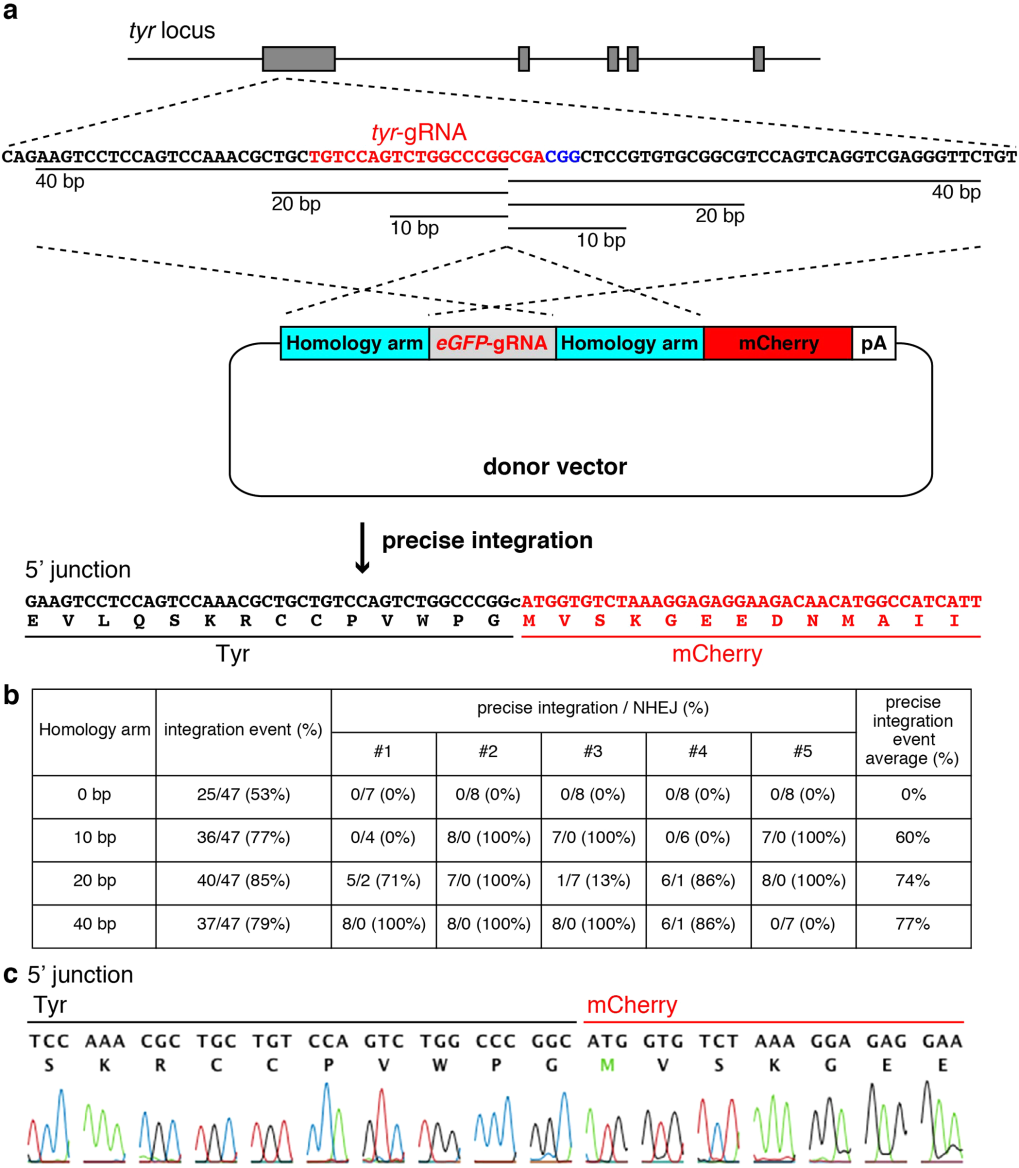
Precise integration of the donor vector into the *tyr* locus. (a) A schematic representation of the *tyrosinase* (*tyr*) locus and the donor vector consisting of the *eGFP*-gRNA target sequence, homology arms, *mCherry* and polyA (pA) signal. The *try*-gRNA target sequences (*tyr*-gRNA site in red, PAM sequence in blue) and the upstream region of *try*-gRNA target sequences were integrated between the *eGFP*-gRNA target sequence and *mCherry* in the donor vector, while the downstream sequences of *try*-gRNA target sequences were integrated into the preceding site of *eGFP*-gRNA target sequences in the donor vector. When the donor vector, gRNAs and Cas9 mRNA were co-injected into 1–2-cell-stage embryos, the *tyr* gene and *mCherry* were connected in the same reading frame by precise integration of the donor vector into the genomic locus. (b) Precise integration of exogenous DNA into the targeted genomic locus. Genomic DNAs were prepared from 47 embryos injected with gRNAs, Cas9 mRNA and the donor vector containing homology arms of different lengths (0 to 40 bp). The integration events were assessed by genomic PCR using primers specific to the genomic locus and donor vector (Supplementary Fig. S1). Sequence analysis was performed in five randomly selected embryos among the integrated individuals to determine whether the donor vectors were integrated homology-dependently. (c) Sequence analysis at the 5′ junction of the genome integrated with the donor vector harbouring homology arms.

DNA double-strand breaks (DSBs) are repaired by MMEJ in addition to NHEJ and HR^24^. We expected that precise genome integration would be enhanced around DSBs by introducing short homology arms into a donor vector. We designed new types of donor vector harbouring homology arms and the gRNA target sequence and investigated whether exogenous DNA was precisely integrated into the zebrafish genome. We chose *tyrosinase*/*tyr* as a targeted genomic locus because the disrupted phenotype of *tyr* is well known as a loss of pigmentation and we had succeeded in designing a good gRNA (*tyr*-gRNA), inducing indel mutations at a rate of 79%^25^. For the donor-vector cleavage we used *eGFP*-gRNA, which induces indel mutations at a rate of 66% and has few off-target effects on the zebrafish genome^21^. In our new donor vector, the sequences flanking the genomic *tyr*-gRNA target site were inserted next to the *eGFP*-gRNA target sequence on the donor vector as short homology arms (Fig. 1a).

When *tyr*-gRNA, *eGFP*-gRNA, Cas9 mRNA and the donor vector without a homology arm (0 bp) were co-injected into 1–2-cell-stage embryos, the donor vector was integrated in the forward direction in 53% of injected embryos (Fig. 1b and Supplementary Fig. S1), presumably through NHEJ-mediated integration of the donor vector into the genomic target site. The introduction of homology arms slightly increased the frequency of integration events (0 bp, 53%, compared with 10–40 bp, 77–85%). To determine whether the integration events were homology-dependent, five embryos were randomly selected, and their sequences at the junction of genome and donor vector were determined. In the case of 10-bp homology arms, 60% of integration events were homology-dependent, and the rest contained various types of indel mutations (Fig. 1b). We observed 74% and 77% of homology-dependent integration in the case of 20-bp and 40-bp homology arms, respectively. Thus, the introduction of homology arms into the donor vector promotes precise integration at the genomic target site (Fig. 1b and Supplementary Fig. S1).

Because the donor vector was designed to connect *tyr* and *mCherry* in the same reading frame, we evaluated the expression of the tyrosinase-mCherry chimeric protein by fluorescent microscopy. However, we observed no mCherry-positive cells in the skin and retina of the injected embryos (Supplementary Fig. S2). The tyrosinase-mCherry chimeric protein may have been non-functional or unstable and degraded immediately, and/or endogenous tyrosinase expression was not sufficient for the detection of the chimeric molecule. We accordingly changed the target gene and improved the donor-vector system.

### Generation of eGFP-tagged Krtt1c19e

**Figure 2:**
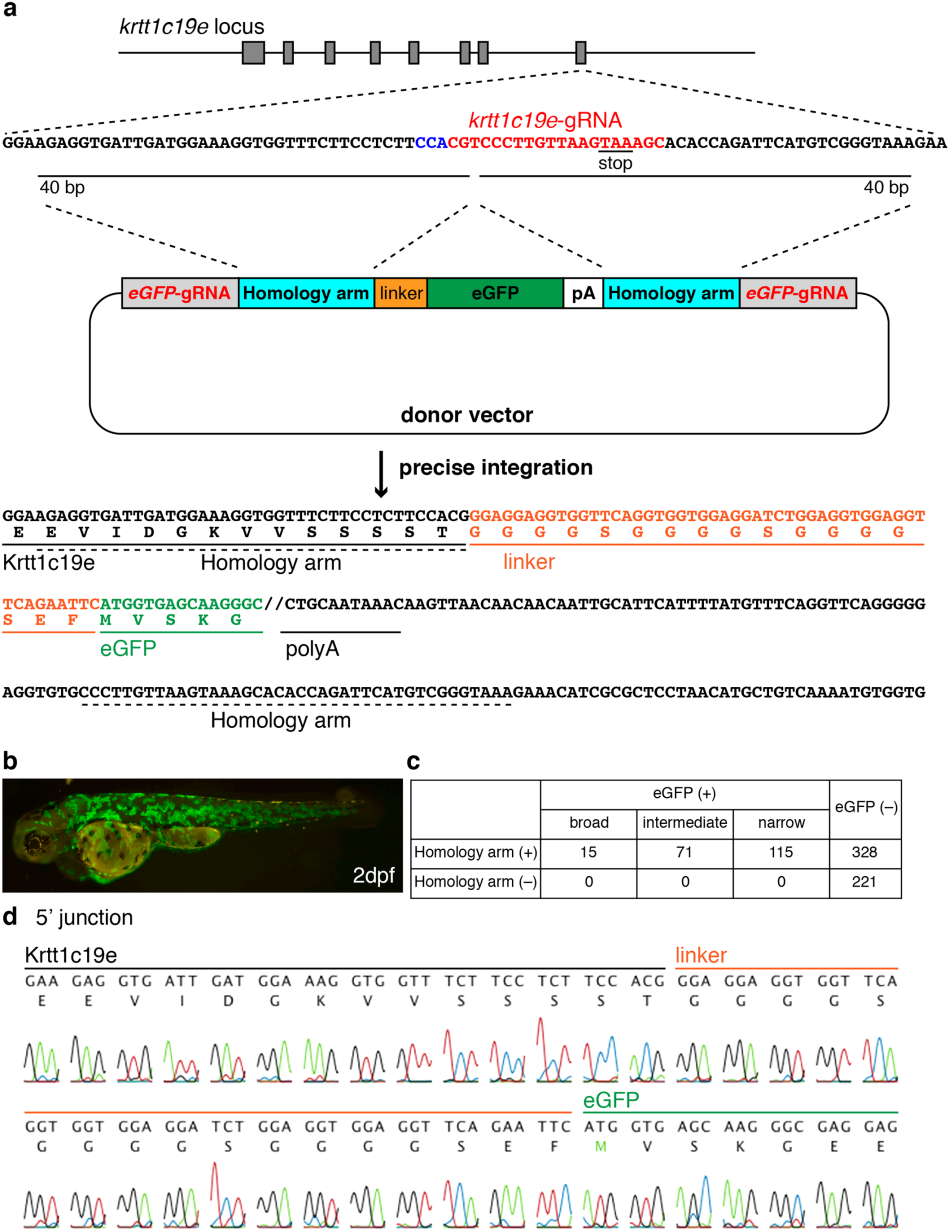
Precise integration of *eGFP* into the *krtt1c19e* locus. (a) A schematic representation of the *krtt1c19e* locus and the donor vector consisting of *eGFP*-gRNA target sequences, homology arms, *eGFP* and polyA (pA) signal. The *krtt1c19e*-gRNA was designed to target the vicinity of the stop codon of the *krtt1c19e* gene. The upstream sequences of the *krtt1c19e*-gRNA target locus (*krtt1c19e*-gRNA sites in red, PAM sequence in blue) were inserted between the *eGFP*-gRNA target sequence and linker sequence on the donor vector, whereas the downstream sequences of the *krtt1c19e*-gRNA target locus were inserted between the polyA signal and *eGFP*-gRNA target sequence in the donor vector. When the donor vector, gRNAs and Cas9 mRNA were co-injected into 1–2-cell-stage embryos, the *krtt1c19e* gene and *eGFP* were connected in the same reading frame via the linker sequence by precise integration into the targeted genomic locus. (b) The injected embryo showed broad eGFP expression in the epidermis 2 days post-fertilisation (dpf). (c) The eGFP expression level was classified into three groups: broad, intermediate and narrow. Representatives of each expression level are shown in Supplementary Fig. S4. We observed no eGFP expression in embryos injected with the donor vector lacking homology arms. (d) Sequence analysis at the 5′ junction of the genome integrated with the donor vector harbouring homology arms.

As the new endogenous target gene, we chose the *keratin type 1 c19e/krtt1c19e* gene, which is highly expressed in basal keratinocytes^26^, and attempted to connect it with eGFP (*eGFP*-gRNA resistance version) at its C-terminus, reasoning that the detection of the chimeric protein would be easier. To tag the C-terminus of Krtt1c19, we designed a *krtt1c19e*-gRNA targeting the vicinity of the stop codon. Injection of *krtt1c19e*-gRNA with Cas9 mRNA into zebrafish embryos induced indel mutations at a rate of 73% (Fig. 2a and Supplementary Fig. S3). We improved the donor vector by introducing the *eGFP* and polyadenylation (polyA) signal sequences between two *eGFP*-gRNA target sequences, for the purpose of eliminating the integration of backbone sequences of the vector. We expected that the DNA fragment harbouring 40-bp homology arms would be precisely integrated into the genomic target locus by homology-dependent integration. We further introduced silent mutations inside the *eGFP* gene to prevent cleavage by the *eGFP*-gRNA.

When the donor vector, *krtt1c19e*-gRNA, *eGFP*-gRNA and Cas9 mRNA were co-injected into 1–2-cell-stage embryos, we observed different degrees of eGFP expression in the epidermis of one-third of the injected embryos (201/529), whereas we observed no embryos expressing eGFP in the absence of the homology arm (Fig. 2b, c and Supplementary Fig. S4). Fifteen of 529 injected embryos exhibited broad eGFP expression in the epidermis of the entire body (Fig. 2b and c). The integration events of the donor vector in the forward direction were detected by PCR analysis in all the embryos expressing eGFP and in three of eight embryos not expressing eGFP (Supplementary Fig. S5). DNA sequence analysis revealed precise integration at both the 5′ and 3′ junctions (Fig. 2d and Supplementary Fig. S6). Furthermore, we analysed another six embryos showing broad eGFP expression by sequencing and confirmed the precise integration at both the 5′ and 3′ junctions in all analysed embryos, although both homology-dependent and -independent integration occurred in two embryos (Supplementary Fig. S7). The integration events of the donor vector in the forward direction were detected by PCR analysis at the 5′ junction in all 16 embryos showing intermediate eGFP expression, whereas they were detected in six of 16 embryos showing narrow eGFP expression (Supplementary Fig. S8). These results suggest that the eGFP expression level in keratinocytes was correlated with the integration frequency.

We further investigated the types of integration events that occurred in the injected embryos (Supplementary Fig. S9). We performed PCR analysis using five primers specific to the genome or to the donor vector. Integration in the opposite direction of the insert fragment (primers 1 and 3) and both directions of the vector fragment (primers 1 and 5, primers 4 and 5) through NHEJ occurred at comparable frequency in the presence or absence of homology arms (Supplementary Fig. S9). Thus, the introduction of homology arms selectively increased by more than twofold the forward-direction integration in the targeted genomic locus (Supplementary Fig. S9). When Cas9 mRNA, *krtt1c19e*-gRNA and the donor vector without *eGFP*-gRNA were co-injected into 1–2-cell-stage embryos, we did not observed integration events (Supplementary Fig. S10), suggesting that the simultaneous cleavage of the donor vector is required for target integration.

### A precise integration event is heritable

**Figure 3:**
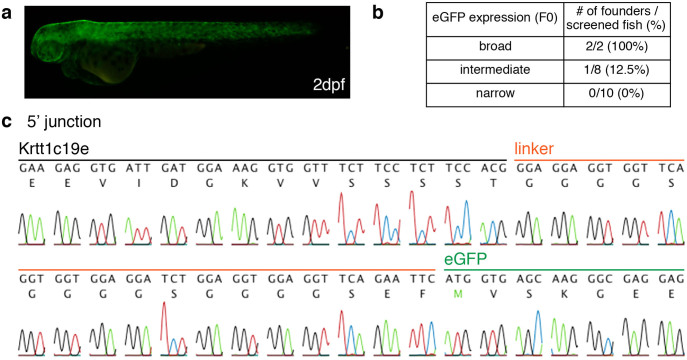
Precise genome modification using CRISPR/Cas9 with our donor vector is heritable. (a) An F1 embryo was obtained by mating wild-type fish with the F0 founder fish injected with *eGFP*-gRNA, *krtt1c19e*-gRNA, Cas9 mRNA and the donor vector, and it exhibited eGFP expression in the epidermis at 2 dpf. (b) The numbers of F0 fish screened and of founders bearing eGFP-positive progeny are indicated. (c) Genomic DNA was prepared from the embryo expressing eGFP, and sequence analysis confirmed the precise integration of eGFP into the *krtt1c19e* locus.

Transmission of the precise genome modification to the germline is a key to establishing a targeted gene knock-in line. To investigate this key process, we crossed potential F0 founders derived from the injected embryos with the wild-type and screened for eGFP expression. Embryos expressing eGFP in their skin were obtained from potential F0 founder fish that exhibited broad eGFP expression in larvae (Fig. 3a and b). When the progeny of the eight potential F0 founder fish that exhibited intermediate eGFP expression in larvae were screened, one positive founder was identified (Fig. 3b). In the case of potential F0 founder fish, which exhibited narrow eGFP expression in larvae, we could not identify a positive founder (Fig. 3b). DNA sequence analysis of eGFP-expressing embryos confirmed the precise integration (Fig. 3c). Germline mosaicisms of F0 founder fish presenting broad eGFP expression in larvae occurred with frequency of 49.5% and 25.3%, while germline mosaicisms of F0 founder fish presenting intermediate eGFP expression in larvae had a frequency of 2.4% (Supplementary Table S2). These data indicate that the pre-screening of injected F0 embryos showing good eGFP expression is important for efficient identification of founder fish.

## Discussion

In this study, we showed that the precise integration of exogenous DNA into the targeted genomic locus in zebrafish can be efficiently achieved using a donor vector containing short homology arms. Concurrent digestion of a donor vector and a targeted genomic locus with artificial site-specific nucleases induces the incorporation of the donor vector into the genomic locus via NHEJ^21^,^22^,^23^,^27^. We improved this method by introducing homology arms into a donor vector. In comparison to ZFNs and TALENs, the CRISPR/Cas9 system is suitable for this method because of the ease of donor-vector construction and of the multiple gRNA design^25^,^28^. The 40-bp homology arms in the donor vector were apparently functional. The use of such short homology arms enabled us to easily construct a donor vector for various target genes; a conventional knock-in vector carries more than 800 bp of homology arms without cleavable sites^19^,^20^.

Recently, Nakade et al. have reported a novel method for achieving precise integration using TALENs and the CRISPR/Cas9 system^29^. In this method, gRNA target sequences in the donor vector are designed to be identical to those flanking the genomic target locus; individual gRNA design is required for each target. In the present study, we designed short homology arms (10–40 bp) for insertion next to gRNA target sequences in the donor vector. Thus, we can use the same gRNA possessing high activity to cleave any donor vector. This feature is advantageous because we can cleave the donor vectors with certainty using already-evaluated gRNAs.

It is thought that the concurrent cleavages of the genome and the donor vector by TALEN or CRISPR/Cas9 are connected by NHEJ. We found that precise genome modification occurred with higher frequency in a homology-dependent manner in our system (Fig. 1b). The donor vector with 40-bp homology arms was integrated in the forward direction in 79% of injected embryos at the *tyr* locus, and precise integration occurred in 77% of these (Fig. 1b), indicating that 61% of injected embryos possess the precisely integrated sequence. This frequency is higher than that of random integration with various indel mutations resulting from the use of donor vectors without homology arms and mediated through NHEJ (53%), suggesting that not only NHEJ but also homology-dependent repair is highly active during early zebrafish development. Furthermore, we confirmed that our method is also functional in human cells. We examined the precise integration of mNeonGreen-2A-puromycin at the human *fibrillarin* (*FBL*) locus in HEK293T cells. After transfection of CRISPR/Cas9 vector and donor vector harbouring 20-bp or 40-bp homology arms, genomic DNAs were prepared from puromycin-resistant cells and the efficient precise integration was confirmed by fluorescence observation and sequencing analysis (Supplementary Fig. S11 and S12). We speculate that MMEJ and/or single-strand annealing (SSA) are involved in these events because of the length of the homology arms. Elucidation of the molecular mechanism behind this method awaits further studies of the mechanism of integration of exogenous DNA into model organisms.

We emphasise that our method enables efficient and precise genome modification in a homology-dependent manner. We have provided evidence that the precise in-frame integration of exogenous DNA from a donor vector carrying short homology arms and cleavage sites is heritable. Thus, our method provides a simple and powerful tool for precise genome modification in zebrafish and mammalian cultured cells.

## Methods

### gRNA vectors and Cas9 plasmid for zebrafish experiments

To construct the vector for synthesising *krtt1c19e*-gRNA, the oligonucleotides (5′-TAGGTTTACTTAACAAGGGACG-3′ and 5′-AAACCGTCCCTTGTTAAGTAAA-3′) were annealed and inserted into *Bsa*I-cleaved pDR274^1^. The vectors for synthesising *eGFP*-gRNA and Cas9 mRNA^30^ were kindly provided by Dr Del Bene (Institut Curie) and Dr Kinoshita (Kyoto University), respectively.

### Construction of donor vectors for zebrafish experiments

To construct donor vectors for targeting the *tyr* locus, *mCherry* was amplified by PCR using the primers mCherry-EcoRI-F and mCherry-XbaI-R (Supplementary Table S1). After *Eco*RI-*Xba*I digestion, the resulting fragment was inserted into the *Eco*RI-*Xba*I-cleaved pCS2P vector from which the CMV promoter was removed by *Hind*III-*Sal*I digestion and blunt-end ligation. To introduce the *eGFP*-gRNA target sequence and homology arm, PCR products using the primers listed in Supplementary Table S1 were phosphorylated and self-ligated.

To construct the donor vectors for targeting the *krtt1c19e* locus, the mutations were introduced in the *eGFP*-gRNA target sequence inside *eGFP* using the primers eGFPmut-F and eGFPmut-R and pCS2P-EGFP^31^ as a template. Then, the linker sequence (GGAGGAGGTGGTTCAGGTGGTGGAGGATCTGGAGGTGGAGGTTCA) was introduced into the 3′ end of *eGFP*. Insert fragments were amplified by PCR using the following primers: krtt1c19e-0bp-F and krtt1c19e-0bp-R for no homology arm; krtt1c19e-40bp-F and krtt1c19e-40bp-R for the 40-bp homology arm. After phosphorylation of insert fragments, the resulting fragments were ligated with the PCR product using the primers eGFP-gRNA-F and eGFP-gRNA-R and pCS2P as a template. The sequences of donor vectors were confirmed by DNA sequencing.

### Preparation of gRNA and Cas9 mRNA

The vectors for *eGFP*-gRNA and *krtt1c19e*-gRNA were digested with *Dra*I and purified by phenol–chloroform extraction and ethanol precipitation. The fragment of *tyr*-gRNA^25^ was prepared by PCR amplification using the primers 5′-GAATTCTAATACGACTCAC-3′ and 5′-AAAAGCACCGACTCGG-3′. PCR products were purified using the MinElute Gel Extraction kit (QIAGEN, Venlo, The Netherlands) after agarose gel electrophoresis. gRNAs were transcribed using the MAXIscript T7 kit (Life Technologies, Carlsbad, CA, USA) and purified by phenol–chloroform extraction and ethanol precipitation. The pCS2+hSpCas9 plasmid was linearised with *Not*I digestion, and Cas9 mRNA was transcribed using the mMESSAGE mMACHINE SP6 kit (Life Technologies) and purified using the RNeasy Mini Kit (QIAGEN).

### Microinjection

The donor vectors, gRNAs and Cas9 mRNA were dissolved in the injection buffer [40 mM HEPES (pH 7.4), 240 mM KCl and 0.5% phenol red] and co-injected into 1–2-cell-stage zebrafish embryos. Each embryo was injected with 1 nl of solution containing 25 ng/μl of each gRNA, 25 ng/μl of donor vector and 250 ng/μl of Cas9 mRNA.

### Preparation of genomic DNA from zebrafish embryos

Genomic DNAs were prepared from uninjected or injected embryos at 2 dpf using the Gentra Puregene Tissue Kit (QIAGEN) according to the manufacturer's instructions. Otherwise, embryos were incubated in 50 μl of lysis buffer [10 mM Tris-HCl (pH 8.0), 1 mM EDTA, 0.2% Triton X-100 and 200 μg/ml proteinase K] at 55°C for 3 h. Then, the solution was incubated at 100°C for 10 min to inactivate proteinase K.

### Detection of donor vector integration into the zebrafish genome

The integration of the donor vector into targeted genomic loci was analysed by PCR with TaKaRa Ex Taq or LA Taq (TaKaRa, Otsu, Japan) using the specific primers listed in Supplementary Table S1. The PCR conditions were as follows: 95°C for 1 min and 30–40 cycles of 98°C for 10 s, 58°C for 30 s and 72°C for 20–75 s. The resulting PCR products were electrophoresed in a 1%, 1.5% or 2% agarose gel. To analyse the sequences, PCR products were subcloned into the pGEM-T Easy vector (Promega, Madison, WI, USA). PCR products from individual colonies using the specific primers listed in Supplementary Table S1 were used directly as templates for sequence analysis.

### Cell culture, transfection and microscopy

Cell culture and transfection were performed as described previously^29^ with some modifications. Briefly, HEK293T cells were maintained in Dulbecco's modified Eagle's medium supplemented with 10% fetal bovine serum. Lipofectamine LTX (Life Technologies) and Opti-MEM (Life Technologies) were used to transfect plasmids, according to the manufacture's instructions. Plasmid concentrations, cell numbers and dishes used were as follows: 1.6 μg for pX330A-*FBL*-2gRNAs CRISPR/Cas9 vector and 0.8 μg for donor vectors into 5.0 × 10^5^ cells using 100-mm dishes. After transfection, cells were cultured in the growth medium described above for three days and then selected with 1 μg/ml puromycin for eight days. For fluorescence observation, cells were moved to glass-bottom plates and fixed with 4% paraformaldehyde in PBS. Cell images were captured with a 488-nm laser using a confocal laser-scanning microscope (Olympus FV-1000D).

### Plasmids for human cell experiments

The Multiplex CRISPR/Cas9 Assembly System Kit (Addgene #1000000055)^32^ was used to construct the all-in-one CRISPR/Cas9 plasmid for human cell expression. Briefly, pX330 vector (Addgene Plasmid 42230) was modified to unify multiple gRNA-expressing cassettes into a single vector. Oligonucleotides for gRNA templates were synthesised, annealed and inserted into the corresponding vectors. Golden Gate assembly was used to assemble the constructed vectors into an all-in-one CRISPR/Cas9 vector for the *FBL* gene, termed pX330A-FBL-2gRNAs, harbouring two gRNA cassettes and a Cas9 cassette. For the donor vectors, gRNA target sequence and 20- or 40-bp homology arms were added in the CRIS-PITCh vector (NCBI accession number LC008488) described previously^29^ using PCR and In-Fusion cloning (Clontech).

### Genomic PCR and DNA sequencing in cultured cells

After puromycin selection, cells were collected and genomic DNA was extracted using a DNeasy Blood and Tissue kit (Qiagen). Genomic PCR was performed using KOD FX Neo (Toyobo) using primers 5′-acaccaagacagacatctctgtcccttg-3′ and 5′-atccgtatccaatgtggggaac-3′ for the 5′ junction, and 5′-gcccttaattgtgagcggataac-3′ and 5′-tcagcaggtcaaggggaggaatg-3′ for the 3′ junction. The PCR products were cloned and transformed into bacteria using a TArget Clone Plus Kit (Toyobo). Subsequently, colony PCR products were used as templates for DNA sequencing.

## Author Contributions

Y.H., T.S., T.Y. and A.K. conceived and designed the work. Y.H. and A.K. wrote the manuscript. Y.H. performed the zebrafish experiments. S.N. performed the human cell experiments. Y.H., S.O., R.O. and A.K. performed data analyses. H.O. gave valuable suggestions. All authors discussed the data. All authors reviewed the manuscript.

## Supplementary Material

Supplementary Information Supplemental Figures
