# Supplementary material for: Precise in-frame integration of exogenous DNA mediated by CRISPR/Cas9 system in zebrafish
Source: Sci Rep. 2015 Mar 5;5:8841. doi: 10.1038/srep08841 (PMC4350073; doi:10.1038/srep08841)
Supplement: Supplementary Information — Supplemental Figures [file srep08841-s1.pdf]

Supplementary Information

Precise in-frame integration of exogenous DNA mediated by CRISPR/Cas9 system in zebrafish

Yu Hisano[1]*, Tetsushi Sakuma[2], Shota Nakade[2], Rie Ohga[3], Satoshi Ota[3], Hitoshi Okamoto[1], Takashi Yamamoto[2], Atsuo Kawahara[3]*

[1]Laboratory for Developmental Gene Regulation, Brain Science Institute, RIKEN, 2-1 Hirosawa, Wako, Saitama, 351-0198, Japan

[2]Molecular Genetics Laboratory, Department of Mathematical and Life Sciences, Graduate School of Science, Hiroshima University, 1-3-1 Kagamiyama, Higashi-Hiroshima, Hiroshima, 739-8526, Japan

[3]Laboratory for Developmental Biology, Center for Medical Education and Sciences, Graduate School of Medical Science, University of Yamanashi, 1110 Shimokato, Chuo, Yamanashi, 409-3898, Japan

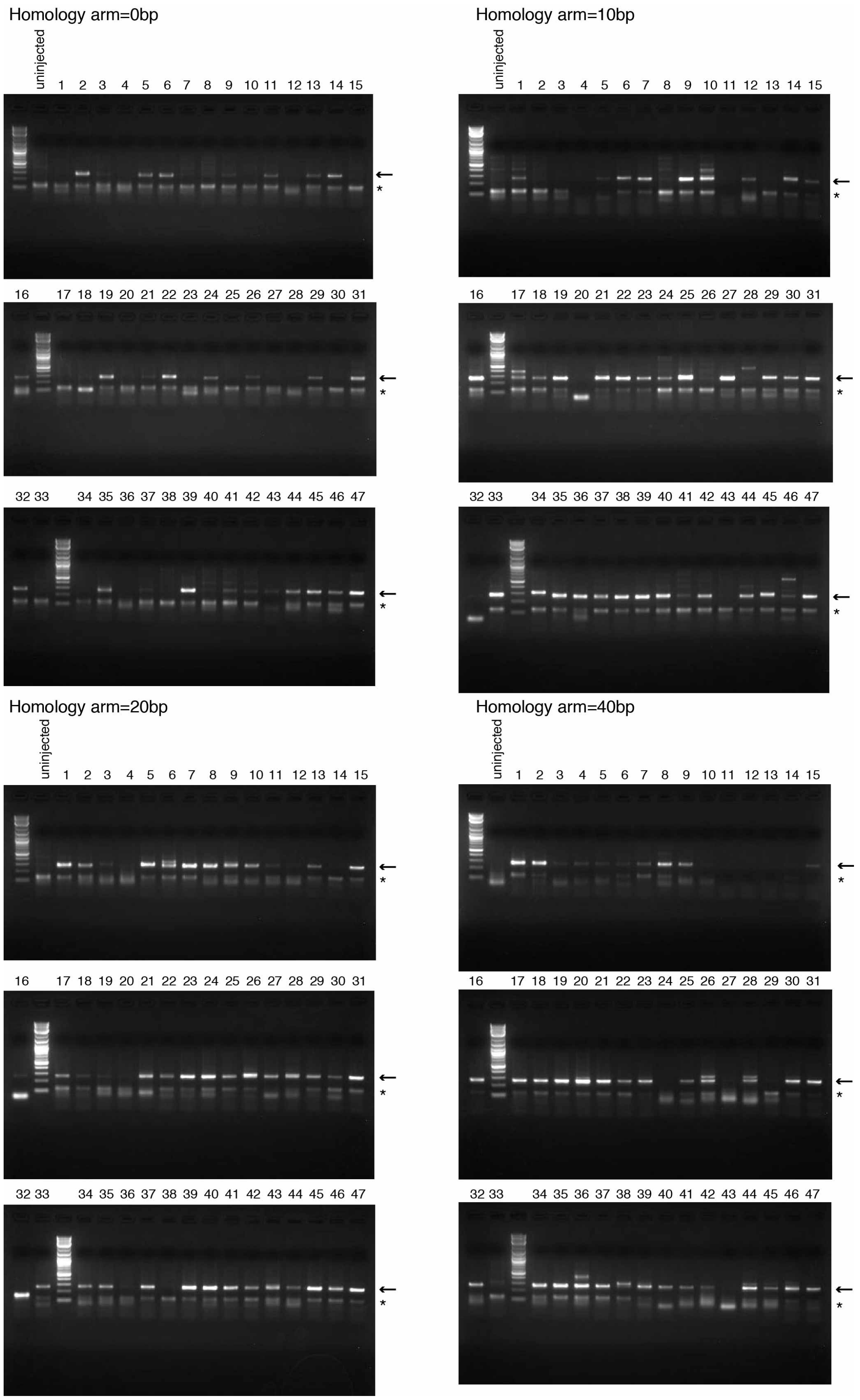
Homology arm=0bp
Homology arm=10bp
Homology arm=20bp
Homology arm=40bp
uninjected
1 2 3 4 5 6 7 8 9 10 11 12 13 14 15
16 17 18 19 20 21 22 23 24 25 26 27 28 29 30 31
32 33 34 35 36 37 38 39 40 41 42 43 44 45 46 47
←
*

Supplementary Figure S1

Genomic PCR to assess the integration events into the *tyr* locus

The genomic DNAs were prepared from 47 embryos injected with *eGFP*-gRNAs, *tyr*-gRNA, Cas9 mRNA and the donor vector containing the homology arms of different lengths (0 bp, 10 bp, 20 bp or 40 bp). PCR products using specific primers, tyr-genome-F and mCherrry-R (Supplementary Table S1), were electrophoresed in a 2% agarose gel. The expected amplicon and non-specific bands (presumably primer dimers) are indicated by arrows and asterisks, respectively.

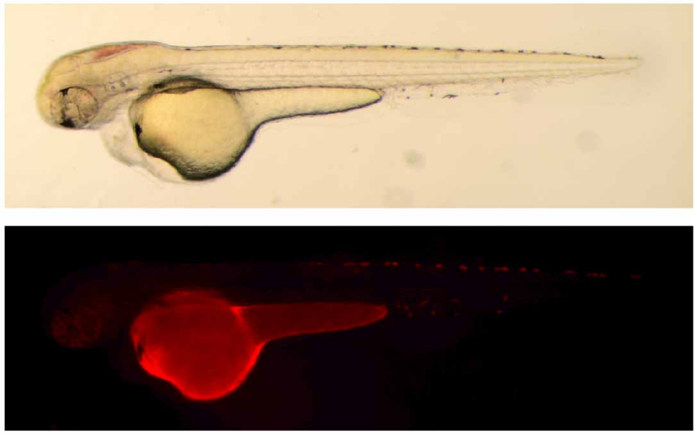

Supplementary Figure S2

Expression of mCherry is not observed in injected embryos

In 2 dpf embryos injected with *eGFP*-gRNA, *tyr*-gRNA, Cas9 mRNA and the donor vector harbouring 40-bp homology arms, mCherry-positive cells were not observed in the skin and retina. Injected embryos exhibited hypopigmentation due to the disruption of the *tyr* gene. Brightfield (upper) and fluorescent (lower) images are of lateral views with dorsal at the top.

krtt1c19e-gRNA (11/15=73%)

```
TTCTTCCTCTTCCACGTCCCTTGTTAAGTAAAGCACACCAGATTC     WT    x4
TTCTTCCTCTTCC-------TTGTTAAGTAAAGCACACCAGATTC     -7    x5
TTCTTCCTCTTCC------CTTGTTAAGTAAAGCACACCAGATTC     -6    x3
TTCTTCCTCTTCCACGT-----GTTAAGTAAAGCACACCAGATTC     -5    x1
TTCTTCCTCTTCCACGTCCCTTGTTAAGTAAAGCACACCAGATTC     +1    x1
                   C
TTCTTCCTCTTCCACGTCCCTTGTTAAGTAAAGCACACCAGATTC     +11   x1
             TCTTCCTCTTC
```

Supplementary Figure S3

Sequence analysis of the *krtt1c19e*-gRNA-induced indel mutations in the target site

The genomic DNAs were prepared from the 2 dpf embryos injected with *krtt1c19e*-gRNA and Cas9 mRNA. The wild-type genomic sequence is shown at the top with the target sequence in red and the PAM sequence in blue. Deleted and inserted nucleotides in the DNA sequences are indicated by dashes and by green letters, respectively. The numbers of deleted (–) and inserted (+) nucleotides are indicated at the right with the detection number.

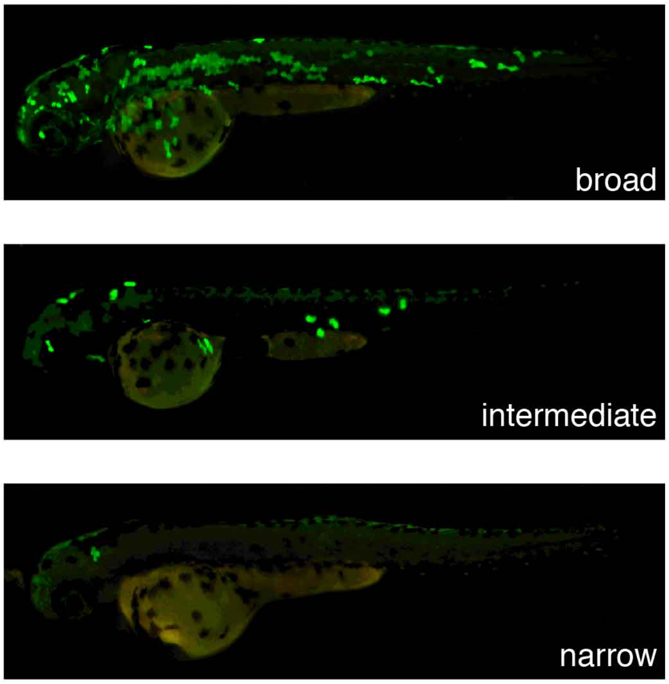

Supplementary Figure S4

The eGFP expression in the embryos injected with the donor vector, gRNAs and Cas9 mRNA

Representatives of individual eGFP expression level (broad, intermediate and narrow) are shown in 2 dpf embryos injected with *eGFP*-gRNA, *krtt1c19e*-gRNA, Cas9 mRNA and the donor vector harbouring homology arms. All images are of lateral views with dorsal at the top.

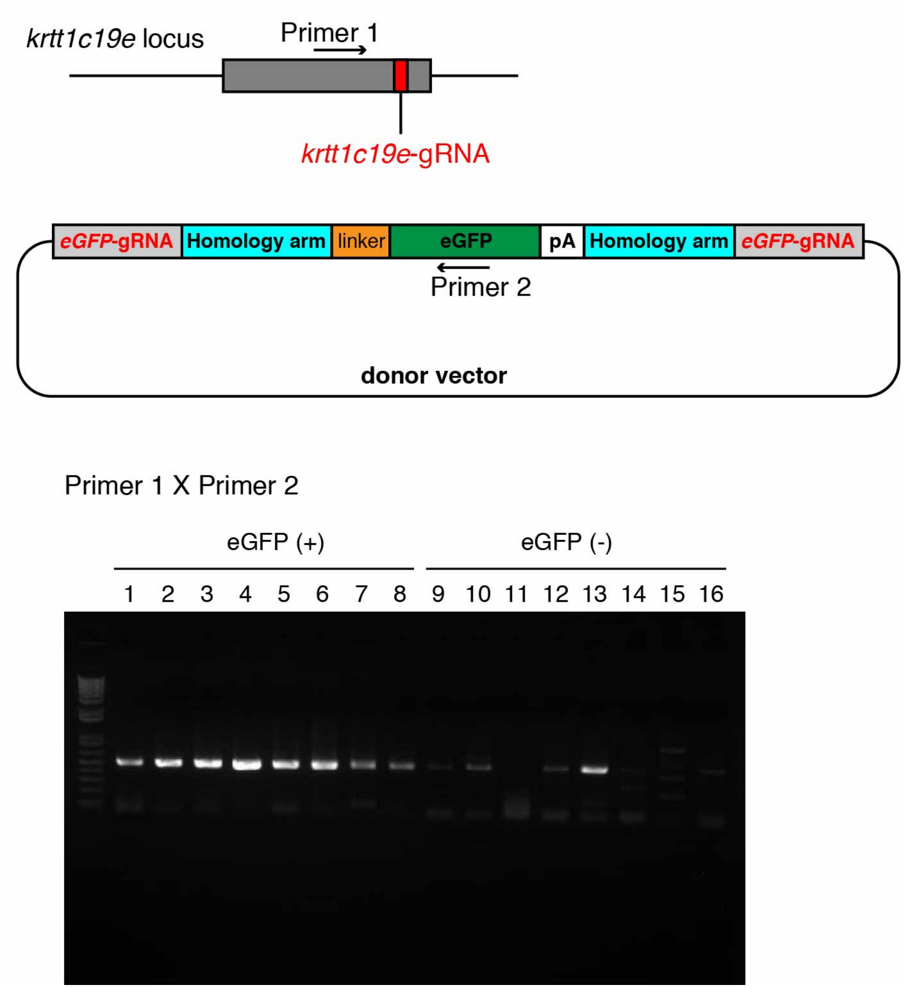

Supplementary Figure S5

Genomic PCR in eGFP-positive or -negative embryos

After the co-injection of *eGFP*-gRNA, *krtt1c19e*-gRNA, Cas9 mRNA and the donor vector harbouring homology arms, the genomic DNAs were prepared from eGFP-positive (+) or -negative (–) embryos. PCR products using the primers specific to the genome (primer 1) and the donor vector (primer 2) were electrophoresed in a 1.5% agarose gel.

3’ junction

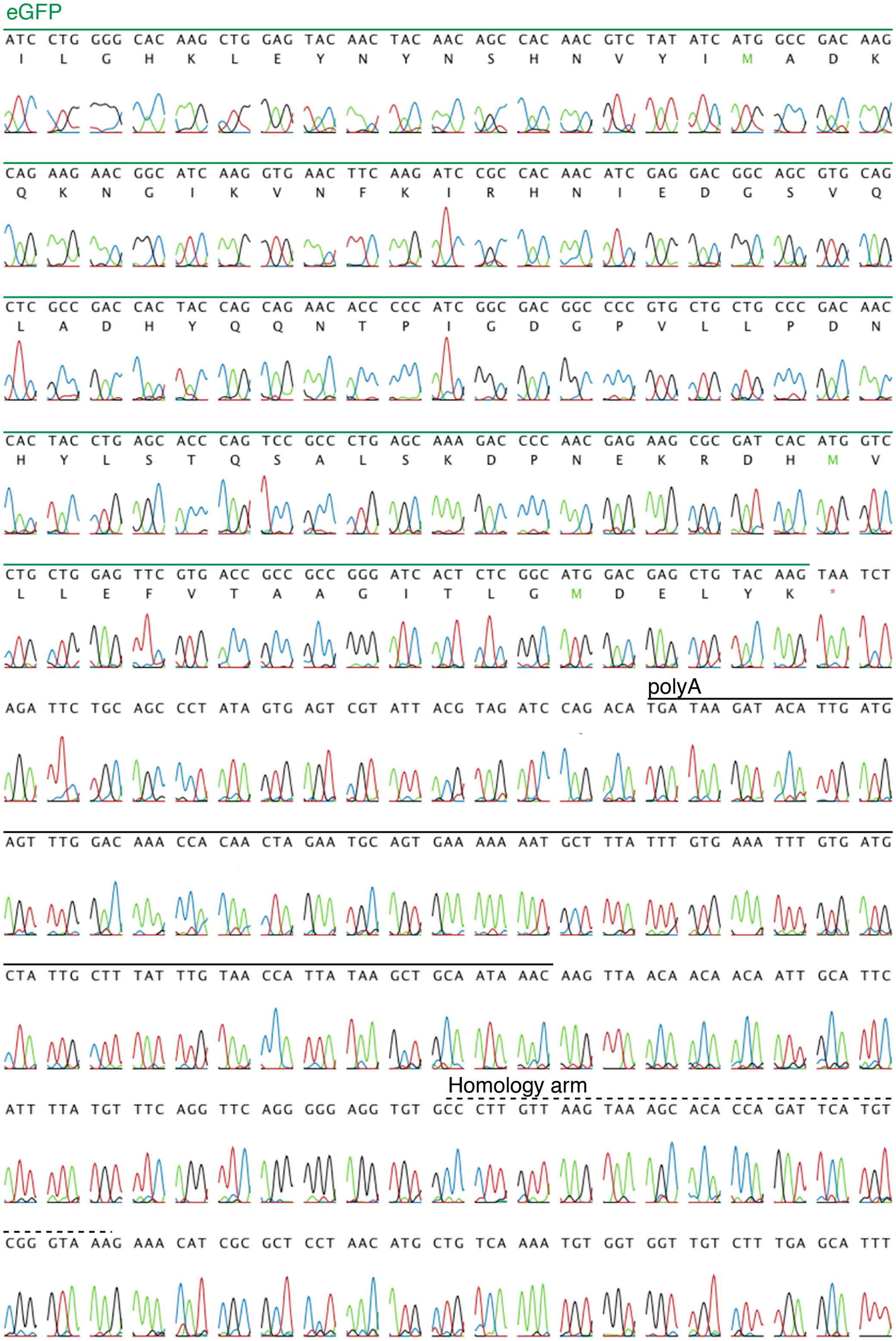
eGFP
ATC CTG GGG CAC AAG CTG GAG TAC AAC TAC AAC AGC CAC AAC GTC TAT ATC ATG GCC GAC AAG
I L G H K L E Y N Y N S H N V Y I M A D K
CAG AAG AAC GGC ATC AAG GTG AAC TTC AAG ATC CGC CAC AAC ATC GAG GAC GGC AGC GTG CAG
Q K N G I K V N F K I R H N I E D G S V Q
CTC GCC GAC CAC TAC CAG CAG AAC ACC CCC ATC GGC GAC GGC CCC GTG CTG CTG CCC GAC AAC
L A D H Y Q Q N T P I G D G P V L L P D N
CAC TAC CTG AGC ACC CAG TCC GCC CTG AGC AAA GAC CCC AAC GAG AAG CGC GAT CAC ATG GTC
H Y L S T Q S A L S K D P N E K R D H M V
CTG CTG GAG TTC GTG ACC GCC GCC GGG ATC ACT CTC GGC ATG GAC GAG CTG TAC AAG TAA TCT
L L E F V T A A G I T L G M D E L Y K *
polyA
AGA TTC TGC AGC CCT ATA GTG AGT CGT ATT ACG TAG ATC CAG ACA TGA TAA GAT ACA TTG ATG
AGT TTG GAC AAA CCA CAA CTA GAA TGC AGT GAA AAA AAT GCT TTA TTT GTG AAA TTT GTG ATG
CTA TTG CTT TAT TTG TAA CCA TTA TAA GCT GCA ATA AAC AAG TTA ACA ACA ACA ATT GCA TTC
Homology arm
ATT TTA TGT TTC AGG TTC AGG GGG AGG TGT GCC CTT GTT AAG TAA AGC ACA CCA GAT TCA TGT
CGG GTA AAG AAA CAT CGC GCT CCT AAC ATG CTG TCA AAA TGT GGT GGT TGT CTT TGA GCA TTT

Supplementary Figure S6

Sequence analysis at the 3′ junction of the donor-integrated genome

Genomic DNA was prepared from the embryos injected with *eGFP*-gRNA, *krtt1c19e*-gRNA, Cas9 mRNA and the donor vector harbouring homology arms. The sequences corresponding to *eGFP* and the polyadenylation (polyA) signal are indicated with solid lines in green and black, respectively.

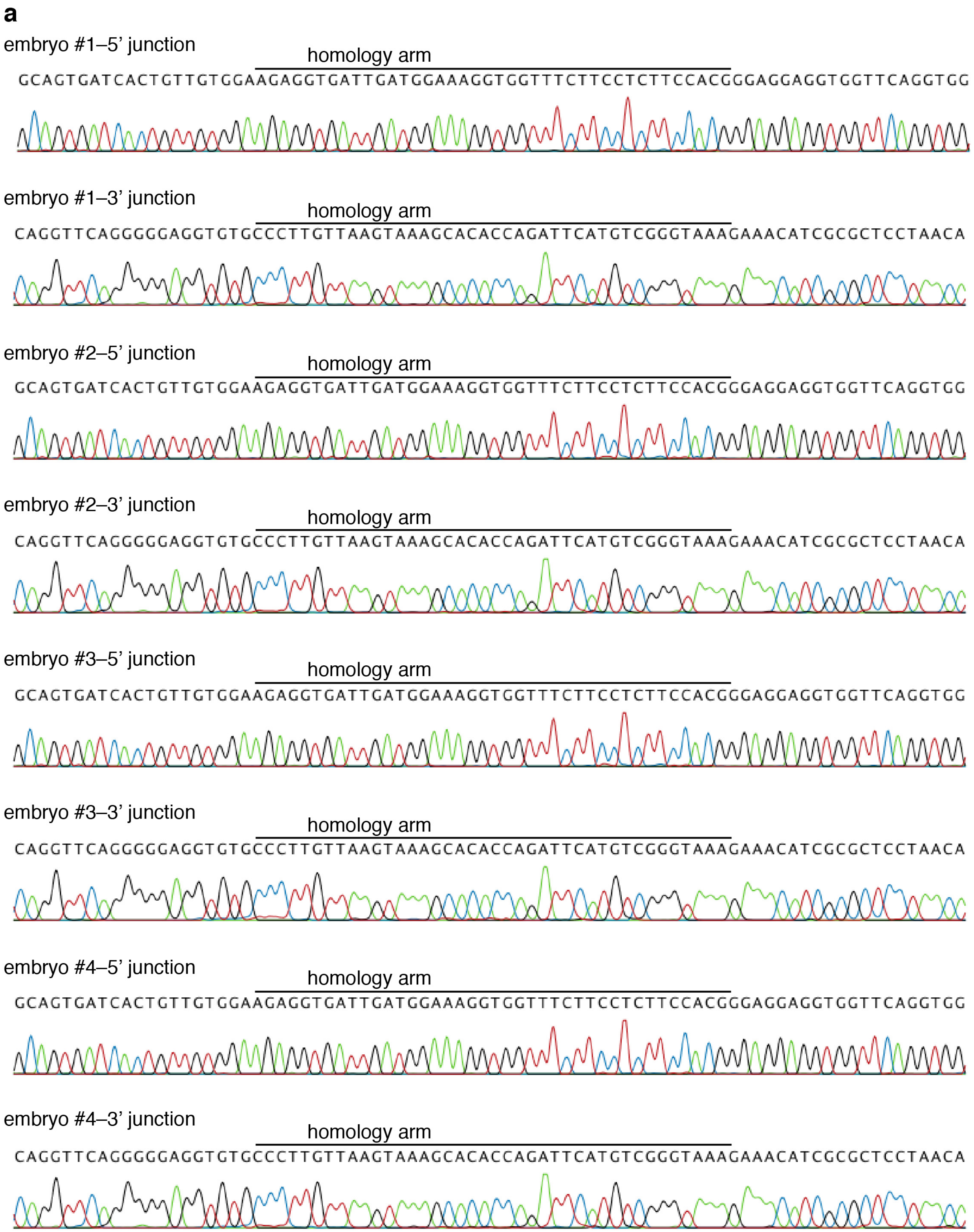
a
embryo #1–5’ junction
homology arm
GCAGTGATCACTGTTGTGGAAGAGGTGATTGATGGAAAGGTGGTTTCTTCCTCTTCCACGGGAGGAGGTGGTTCAGGTGG
embryo #1–3’ junction
homology arm
CAGGTTCAGGGGGAGGTGTGCCCTTGTTAAGTAAAGCACACCAGATTCATGTCGGGTAAAGAAACATCGCGCTCCTAACA
embryo #2–5’ junction
homology arm
GCAGTGATCACTGTTGTGGAAGAGGTGATTGATGGAAAGGTGGTTTCTTCCTCTTCCACGGGAGGAGGTGGTTCAGGTGG
embryo #2–3’ junction
homology arm
CAGGTTCAGGGGGAGGTGTGCCCTTGTTAAGTAAAGCACACCAGATTCATGTCGGGTAAAGAAACATCGCGCTCCTAACA
embryo #3–5’ junction
homology arm
GCAGTGATCACTGTTGTGGAAGAGGTGATTGATGGAAAGGTGGTTTCTTCCTCTTCCACGGGAGGAGGTGGTTCAGGTGG
embryo #3–3’ junction
homology arm
CAGGTTCAGGGGGAGGTGTGCCCTTGTTAAGTAAAGCACACCAGATTCATGTCGGGTAAAGAAACATCGCGCTCCTAACA
embryo #4–5’ junction
homology arm
GCAGTGATCACTGTTGTGGAAGAGGTGATTGATGGAAAGGTGGTTTCTTCCTCTTCCACGGGAGGAGGTGGTTCAGGTGG
embryo #4–3’ junction
homology arm
CAGGTTCAGGGGGAGGTGTGCCCTTGTTAAGTAAAGCACACCAGATTCATGTCGGGTAAAGAAACATCGCGCTCCTAACA

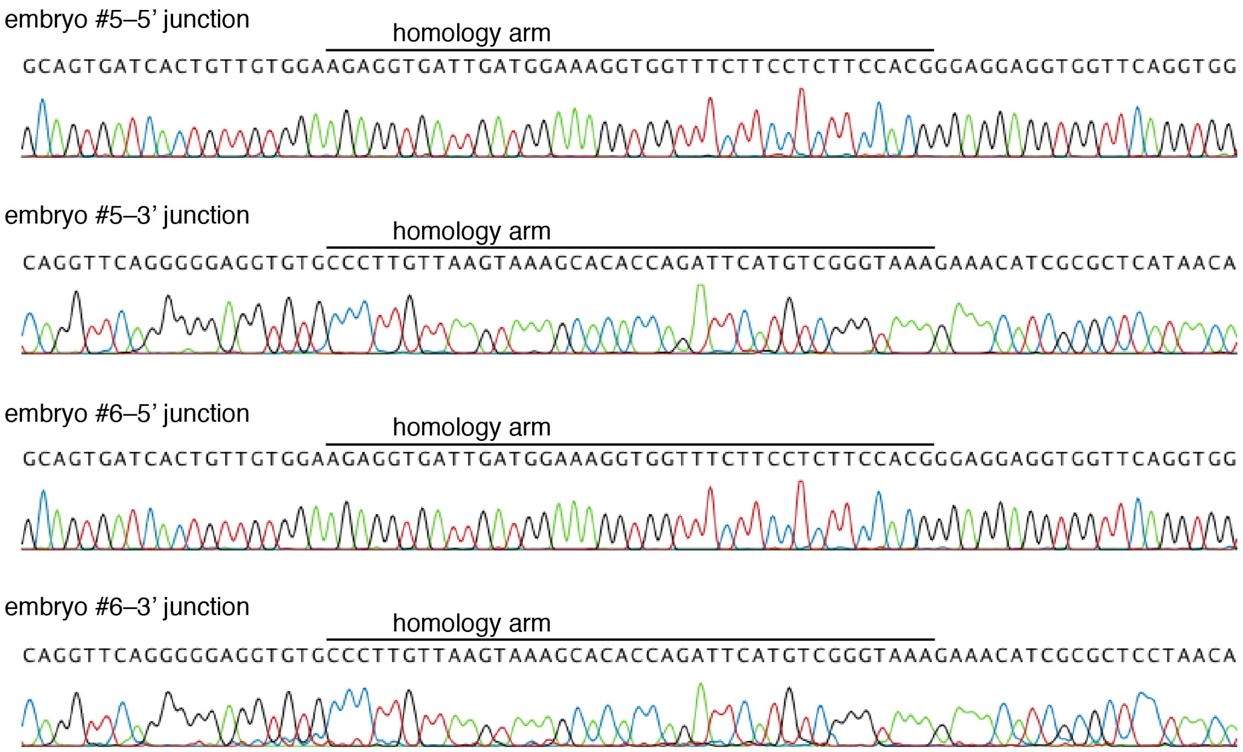

b

	precise integration / NHEJ					
	#1	#2	#3	#4	#5	#6
5' junction	2/6	6/0	6/0	7/0	5/0	7/0
3' junction	8/0	5/3	7/0	8/0	8/0	8/0

Supplementary Figure S7

Sequence analysis of the embryos broadly expressing eGFP

(a) After the co-injection of *eGFP*-gRNA, *krtt1c19e*-gRNA, Cas9 mRNA and the donor vector harbouring homology arms into zebrafish embryos, the genomic DNAs were prepared from six embryos showing broad eGFP expression. Sequence analysis was performed to examine the 5′ and 3′ junction of the genome integrated with the donor vector harbouring homology arms. The sequences corresponding to the homology arm are indicated with solid lines in black. (b) Several clones (5–8) derived from individual embryos were analysed. The numbers of clones in which the donor vector was integrated homology-dependently (precise integration) and -independently (NHEJ) are indicated.

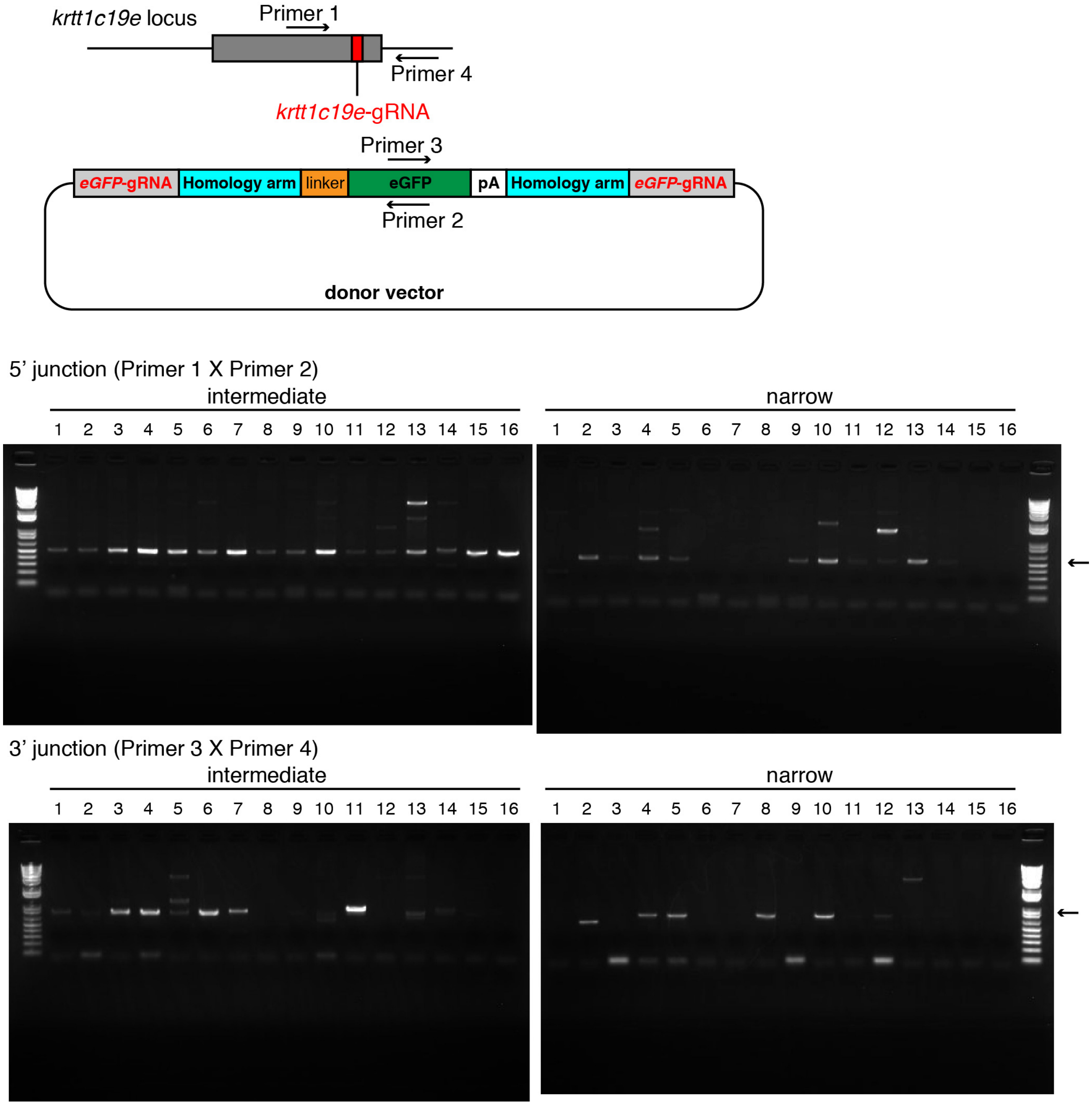

Supplementary Figure S8

Genomic PCR in the injected embryos expressing eGFP at intermediate or narrow levels

After the co-injection of *eGFP*-gRNA, *krtt1c19e*-gRNA, Cas9 mRNA and the donor vector harbouring homology arms into zebrafish embryos, the genomic DNAs were prepared from embryos showing intermediate or narrow eGFP expression. PCR products using the primers specific to the genome (primer 1 or 4) and the donor vector (primer 2 or 3) were electrophoresed in a 1.5% agarose gel. The expected amplicon bands are indicated by arrows.

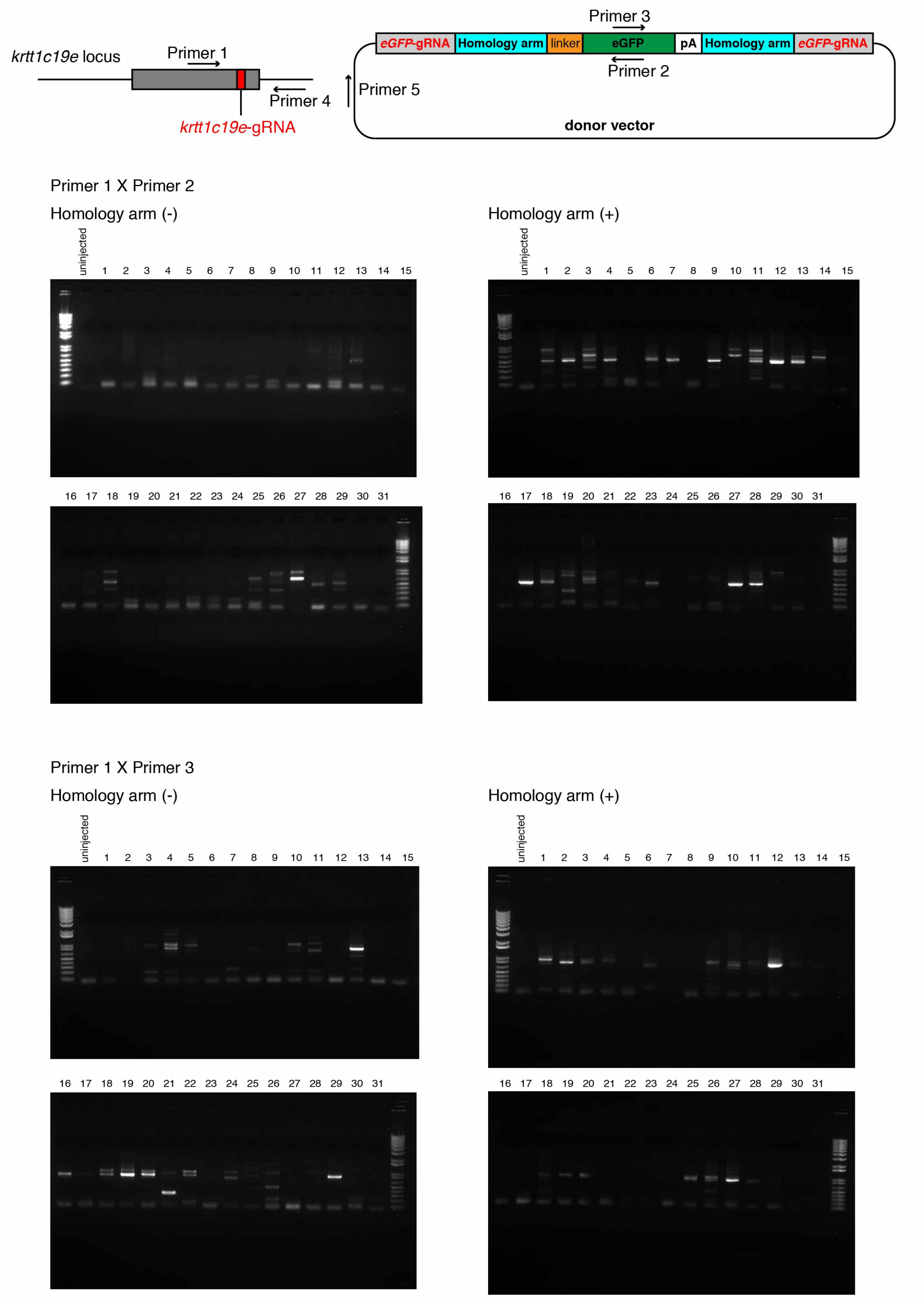

krtt1c19e locus
Primer 1
Primer 4
krtt1c19e-gRNA
Primer 3
eGFP-gRNA
Homology arm
linker
eGFP
pA
Homology arm
eGFP-gRNA
Primer 2
Primer 5
donor vector
Primer 1 X Primer 2
Homology arm (-)
Homology arm (+)
uninjected
1 2 3 4 5 6 7 8 9 10 11 12 13 14 15
16 17 18 19 20 21 22 23 24 25 26 27 28 29 30 31
Primer 1 X Primer 3
Homology arm (-)
Homology arm (+)
uninjected
1 2 3 4 5 6 7 8 9 10 11 12 13 14 15
16 17 18 19 20 21 22 23 24 25 26 27 28 29 30 31

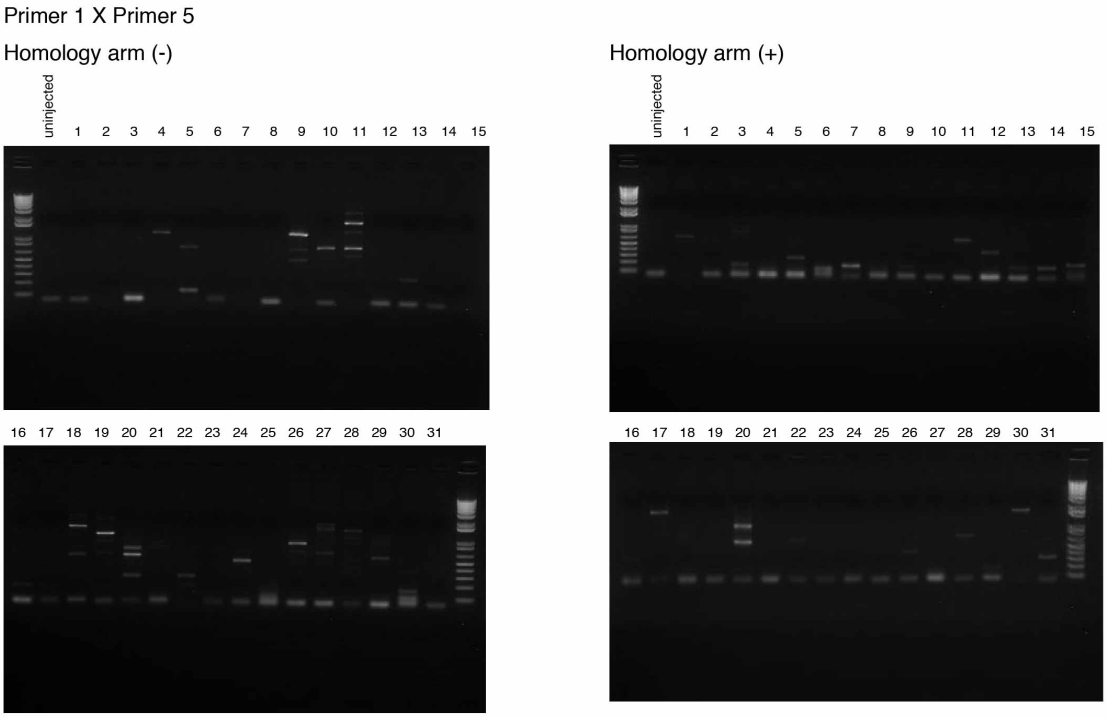

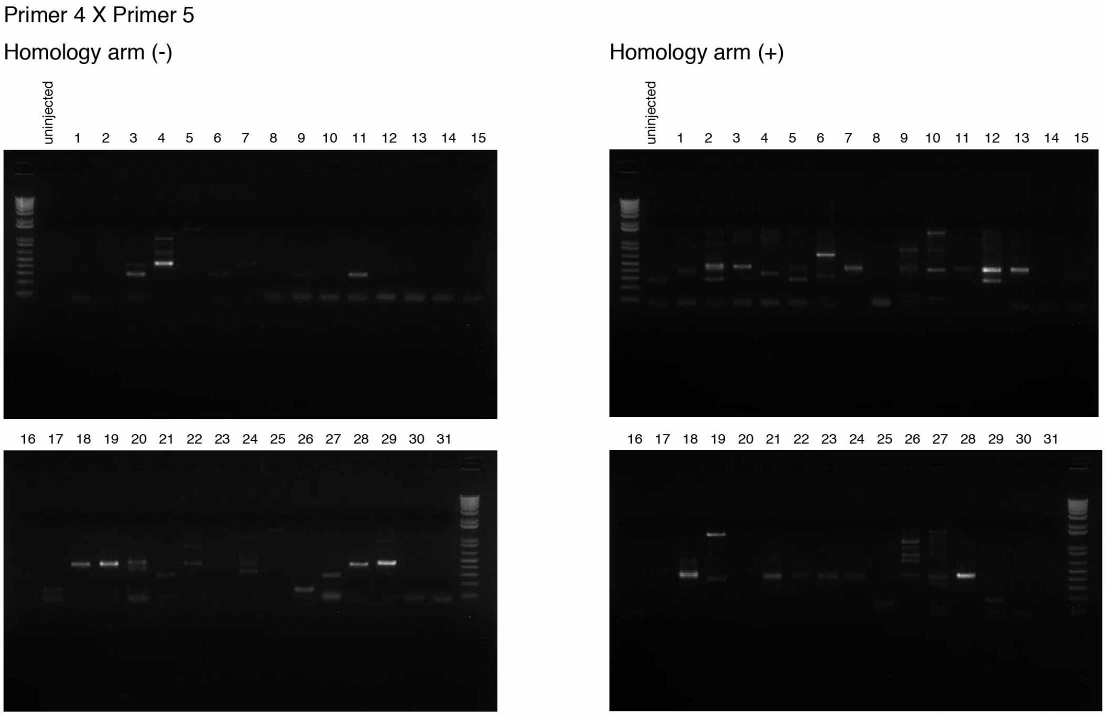

	Integration event (%)			
Primer pair	Primer 1	Primer 1	Primer 1	Primer 4
	Primer 2	Primer 3	Primer 5	Primer 5
Homology arm (+)	19/31 (61%)	15/31 (48%)	7/31 (23%)	16/31 (52%)
Homology arm (-)	7/31 (23%)	13/31 (32%)	14/31 (45%)	12/31 (39%)

Supplementary Figure S9

Genomic PCR to assess the integration events into the *krtt1c19e* locus

Five primers specific to the genome or the donor vector were designed to assess the integration events in the absence or presence of homology arms. The genomic DNAs were prepared from 31 embryos injected with *eGFP*-gRNA, *krtt1c19e*-gRNA, Cas9 mRNA and the donor vector harbouring or lacking homology arms. Primers 1 and 2 were used to detect the insert fragment in the forward direction. Primers 1 and 3 were used to detect the insert fragment in the reverse direction. Primers 1 and 5 were used to detect the vector fragment in the forward direction. Primers 4 and 5 were used to detect the vector fragment in the reverse direction. The results of integration events are shown in the Table.

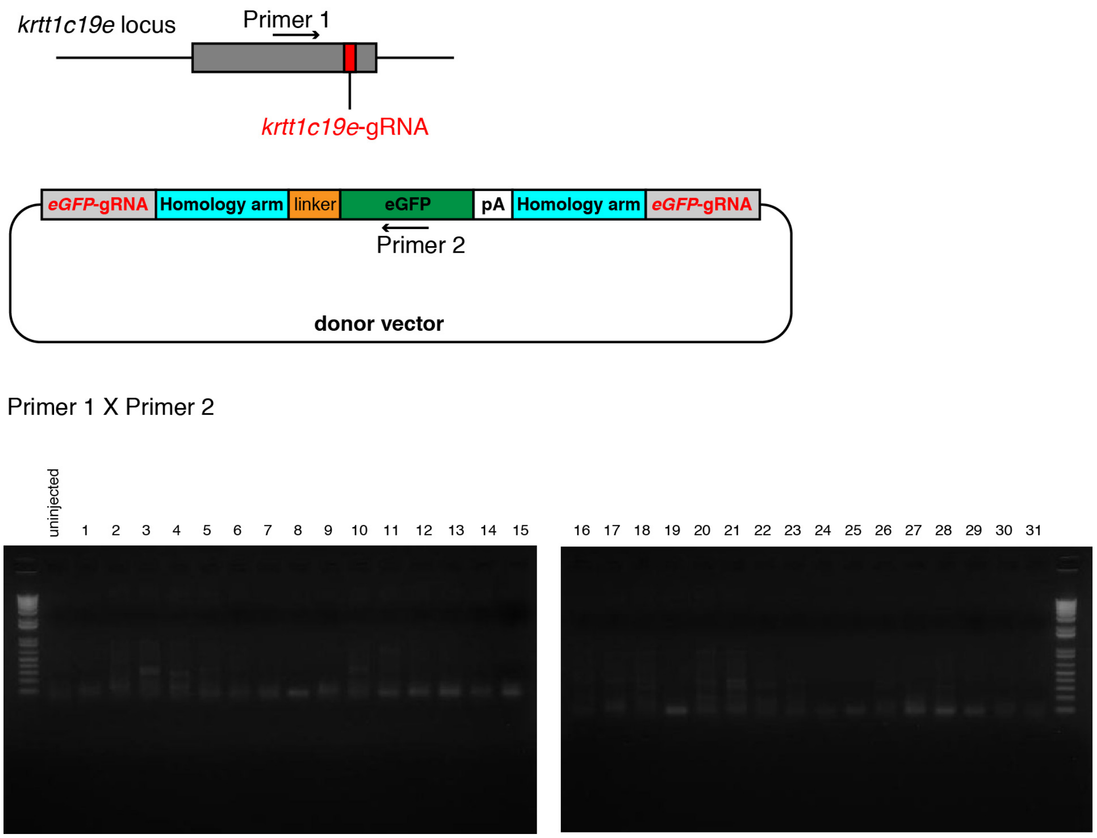

Supplementary Figure S10

Genomic PCR in the embryos injected with the donor vector, *krtt1c19e*-gRNA and Cas9 mRNA, but not *eGFP*-gRNA

After the co-injection of *krtt1c19e*-gRNA, Cas9 mRNA and the donor vector harbouring homology arms into zebrafish embryos, the genomic DNAs were prepared from embryos. PCR products using the primers specific to the genome (primer 1) and the donor vector (primer 2) were electrophoresed in a 1.5% agarose gel. We could not detect the integration of the donor vector in 31 embryos that we examined.

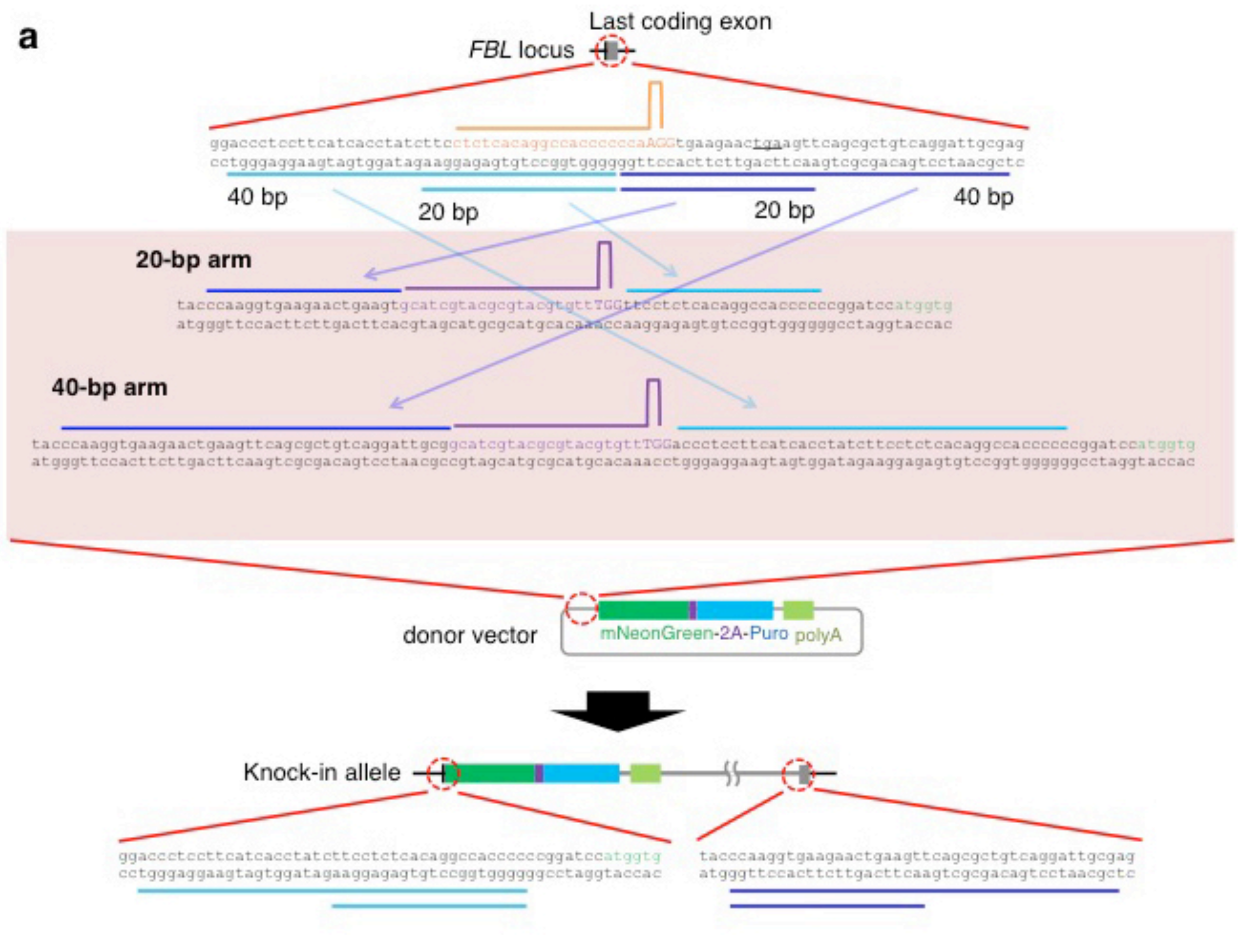

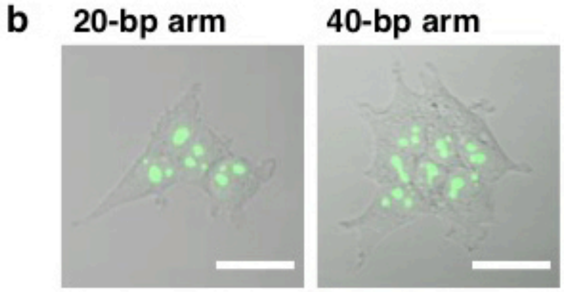

c

	5' junction	3' junction
**20-bp arm**	14/16 (87.5%)	9/15 (60.0%)
**40-bp arm**	10/16 (62.5%)	12/15 (80.0%)

Supplementary Figure S11

Precise gene knock-in in human cells

(a) Schematic illustration of CRISPR/Cas9-mediated targeted integration at the *fibrillarin* (*FBL*) locus in human cells. Orange and purple letters indicate the gRNA target sites. Light blue and blue bars indicate the homology arms. The stop codon is underlined. (b) Confocal laser scanning microscopy images of cells after puromycin selection. Scale bar: 30 μm. (c) Numbers and percentages of precisely knocked-in clones. Correct knock-in was confirmed by DNA sequencing (Supplementary Figure S12).

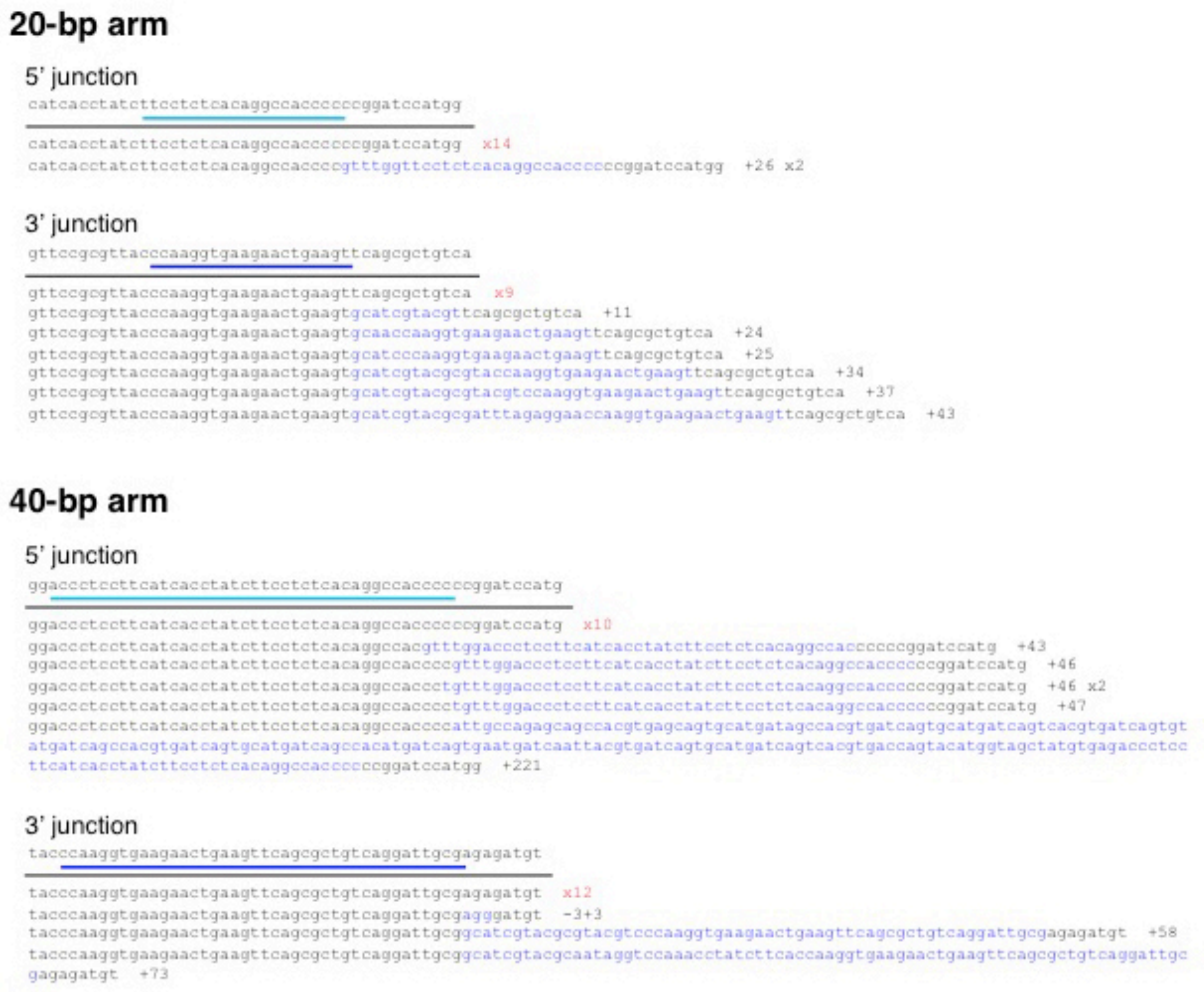

Supplementary Figure S12

Sequences of knocked-in alleles

The intended knocked-in sequence is shown at the top of each panel. Light blue and blue bars indicate the homology arms. Red letters indicate precisely knocked-in alleles. Blue letters indicate insertions.

Supplementary Table S1.

Primers used in this study.

PCR primers used for detecting integration of donor plasmid into the *tyr* locus

Primer name	Sequence (5′ to 3′)
tyr-genome-F	GAGTCTGCACCTCCCCAGAAGTC
mCherrry-R	GACAGAATGTCCCATGCGAAAGG

PCR primers used for detecting integration of donor plasmid into the *krtt1c19e* locus

Primer name	Sequence (5′ to 3′)
primer 1/krtt1c19e-genome-F	CAGCCTTGCAATTTCTAGCAAATCG
primer 2/eGFP-R	GTCGTCCTTGAAGAAGATGGTGC
primer 3/eGFP-F	GCACCATCTTCTTCAAGGACGAC
primer 4/krtt1c19e-genome-R	ACACACATTCTGTCTTTGACGTTAGC
primer 5/vector-F	GCCGTAAAGCACTAAATCGGAACC

Primers used for colony PCR of the pGEM-T Easy vector

Primer name	Sequence (5′ to 3′)
colonyPCR-F	AGCTCACTCATTAGGCAC
colonyPCR-R	GTAAAACGACGGCCAGT

Primers used for constructing donor plasmids

Primer name	Sequence (5′ to 3′)
mCherry-EcoRI-F	CCTGAATTCATGGTGTCTAAAGGAGAGGAAGAC
mCherry-XbaI-R	TAGTCTAGATCACTTATACAGCTCGTCCATGC
tyr-0bp-F	TGCCACCTACGGCATGGTGTCTAAAGGAGAGGAAGAC
tyr-0bp-R	TCGCCCTCGCCGTCACCTAAATCAAGCTTCGACTGG
tyr-10bp-F	TGCCACCTACGGTCTGGCCCGGCATGGTGTCTAAAGGAGAGGAAGAC
tyr-10bp-R	TCGCCCTCGCCGGAGCCGTCGGTCACCTAAATCAAGCTTCGACTGG
tyr-20bp-F	TGCCACCTACGGTGCTGTCCAGTCTGGCCCGGCATGGTGTCTAAAGGAGAGGAAGAC
tyr-20bp-R	TCGCCCTCGCCCGCCGCACACGGAGCCGTCGGTCACCTAAATCAAGCTTCGACTGG
tyr-40bp-F	TGCCACCTACGGAAGTCCTCCAGTCCAAACGCTGCTGTCCAGTCTGGCCC
tyr-40bp-R	TCGCCCTCGCCGAACCCTCGACCTGACTGGACGCCGCACACGGAGCCGTCG
eGFPmut-F	CTACATACGGCAAGCTGACCCTGAAGTTCA
eGFPmut-R	CGTCCCCTTCGCCCTCGCCGGACACGC
krtt1c19e-0bp-F	GGAGGAGGTGGTTCAGGTGG
krtt1c19e-0bp-R	CACACCTCCCCCTGAACCTG
krtt1c19e-40bp-F	AGAGGTGATTGATGGAAAGGTGGTTTCTTCCTCTTCCACGGGAGGAGGTGGTTCAGGTGG
krtt1c19e-40bp-R	TTTACCCGACATGAATCTGGTGTGCTTTACTTAACAAGGGCACACCTCCCCCTGAACCTG
eGFP-gRNA-F	CCGTAGGTGGCATCGCCCTCGCCTCGACTGGCGTAATAGCGAAGAG
eGFP-gRNA-R	CCGTAGGTGGCATCGCCCTCGCCAGTGAGGGTTAATTGCGCGC

Supplementary Table S2

Germline transmission of eGFP knock-in line

Founder	F1 individuals evaluated	Positive individuals	% of eGFP-positive F1 embryos
Founder #1 (broad)	97	48	49.5%
Founder #2 (broad)	360	91	25.3%
Founder #3 (intermediate)	165	4	2.4%
